# Automated quantitative analysis can support the identification of progressive pulmonary fibrosis in fibrotic interstitial lung disease

**DOI:** 10.1136/bmjresp-2026-004217

**Published:** 2026-10-20

**Authors:** Emma Janska, Giles Dixon, Joseph Page, Tom M Hartley, Sharon C Sturney, James C Harper, Krasimira Tsaneva-Atanasova, Michael A Gibbons, Shaney L Barratt, Chris J Scotton, Jonathan C L Rodrigues

**Affiliations:** 1 University of Bristol Medical School, Bristol, UK; 2 Royal United Hospitals Bath NHS Foundation Trust, Bath, UK; 3 Department of Clinical & Biomedical Sciences, University of Exeter, Exeter, UK; 4 Department of Respiratory Medicine, Royal United Hospitals Bath NHS Foundation Trust, Bath, UK; 5 Department of Mathematics and Statistics, University of Exeter, Exeter, UK; 6 Living Systems Institute, University of Exeter, Exeter, UK; 7 Academic Department of Respiratory Medicine, Royal Devon University Healthcare NHS Foundation Trust, Exeter, UK; 8 NIHR Exeter Biomedical Research Centre, University of Exeter, Exeter, UK; 9 Bristol Interstitial Lung Disease Service, North Bristol NHS Trust, Bristol, UK; 10 Academic Respiratory Unit, University of Bristol, Bristol, UK; 11 Radiology, Royal United Hospitals Bath NHS Foundation Trust, Bath, UK; 12 Department of Health, University of Bath, Bath, UK

**Keywords:** Interstitial Fibrosis, Imaging/CT MRI etc

## Abstract

**Background:**

Identifying progression in fibrotic interstitial lung disease (f-ILD) is essential for treatment decisions and prognostication. Quantitative CT (qCT) may improve detection of progressive pulmonary fibrosis (PPF). We evaluated whether routine qCT enhances identification of disease progression.

**Methods:**

We conducted a retrospective observational study of patients presenting to an ILD service over 2 years. Patients with f-ILD who underwent two CT scans within 24 months prior to ILD multidisciplinary team (ILD-MDT) review were included. CTs were initially reported by a mixed pool of radiologists and subsequently reviewed by an expert thoracic radiologist at ILD-MDT. The contextflow ADVANCE Chest CT was used to quantify lung abnormalities.

The cohort was divided 70:30 into derivation and validation sets, stratified by ILD-MDT-defined progression. In the derivation cohort, optimal qCT thresholds were defined using Youden’s index and applied to the validation cohort to assess detection of previously unreported progression.

**Results:**

127/502 patients met inclusion criteria and were stratified into derivation (n=89) and validation (n=38) cohorts. In the derivation cohort, a 0.39% increase in combined honeycombing and reticulation yielded an AUC of 0.64 for identifying progression.

In the validation cohort, this threshold achieved sensitivity and specificity of 0.71 for PPF detection. Although 27/38 (71.1%) patients were initially reported as stable or without comment on progression, 12/27 (44.4%) were later classified as having radiological progression at ILD-MDT. Of these discordant cases, qCT correctly identified progression in 10/12 (83.3%).

**Discussion:**

This study demonstrates that qCT may assist the non-specialist radiologist in identifying fibrotic disease progression enabling earlier access to appropriate treatment.

**WHAT IS ALREADY KNOWN ON THIS TOPIC**

- Quantitative CT has been extensively reported demonstrating correlation with pulmonary function testing and association with progression- and transplant-free survival. There has been little study demonstrating potential clinical adoption strategies to bring patient benefit from these algorithms.

**WHAT THIS STUDY ADDS**

- This study provides a proof of concept framework for the adoption of qCT into fibrotic-ILD patient pathways. The study demonstrates the serial automated quantification of honeycombing and reticulation quantification could reduce the number of high-resolution CT scans incorrectly labelled as stable or with no statement on disease progression.

**HOW THIS STUDY MIGHT AFFECT RESEARCH, PRACTICE OR POLICY**

- This study provides the evidence to commence a prospective, multicentre adoption and validation study to bring patient benefit from quantitative CT.

## Introduction

 Interstitial lung disease (ILD) describes a group of over 200 conditions resulting in disruption to the lung parenchyma with thickening of the lung interstitium and compromised gas exchange.^1^ ILD is associated with significant mortality with life expectancy for common ILDs being almost half of the general population.^2^

Progressive pulmonary fibrosis (PPF) describes a cohort of fibrotic ILD patients in whom their disease has progressed despite optimal management.^3^ Depending on the subtype of ILD, between 40% and 60% of all patients may develop PPF.^4^ Patients with PPF are now eligible for treatment with the antifibrotic drug nintedanib in the UK following NICE TA747 which acknowledged evidence from the INBUILD trial.^5 6^ The landmark INBUILD trial defined PPF as a combination of worsening pulmonary physiology, increasing fibrosis on CT scan or worsening pulmonary symptoms. This study has highlighted the need to robustly identify disease progression in patients with non-IPF ILD. Furthermore, as other disease-modifying treatments become available for patients with idiopathic pulmonary fibrosis (IPF) and PPF, identifying progression to enable treatment modification will become vital in management of ILD.

CT scans are routinely reported by both expert and general radiologists, the gold standard for the diagnosis of radiological progression is the use of a specialist ILD-multidisciplinary team meeting (ILD-MDT) with expert thoracic radiologist interpretation. Resource and practical limitations mean that not all serial CT scans performed in patients with fibrotic ILD can be discussed at ILD-MDT. Quantitative computed tomography (qCT) is the use of algorithms to derive radiomic biomarkers of disease from CT scans which can be used for prognostication and disease monitoring.^7^ Although the prognostic performance of these algorithms has been widely reported, their potential impact on clinical decision-making and patient pathways has not been well characterised.

We aimed to investigate the use of qCT to assist the non-specialist radiologist to identify potential PPF that could then be referred for specialist assessment at ILD-MDT.

## Methods

### Study design

This was a retrospective, observational, single-centre study conducted at the Royal United Hospitals Bath NHS Foundation Trust, UK. Patients discussed at the weekly ILD-MDT meeting between 01/01/2023 and 31/12/2024 were screened for inclusion. The study evaluated whether automated quantitative CT (qCT) analysis could assist non-specialist radiologists in identifying radiological fibrotic disease progression on serial CT imaging.

### Eligibility

Patients were eligible if they had a diagnosis of interstitial lung abnormality or fibrotic ILD and had undergone at least two thoracic CT scans (contrast or non-contrast) within the 24 months preceding MDT discussion. 24 months was chosen to reflect current UK prescribing criteria for the antifibrotic drug nintedanib for PPF. Where patients were discussed more than once, only the first MDT discussion was included. Cases were included only if an ILD-MDT thoracic radiologist classified disease status as either stable or progressive; cases without a definitive expert classification were excluded.

### Baseline data

Participant demographics, clinical working diagnosis, radiological diagnosis and clinical features were recorded. Only patients with successful automated qCT processing of both CT scans were included in the final analysis. qCT analysis was performed in retrospect, and processing times were approximately 10 min per scan.

### PPF criteria

Pulmonary function tests were retrospectively analysed against INBUILD criteria for PPF. Electronic patient records were reviewed to identify progressive symptoms over the prior 2 years including increasing breathlessness, cough or fatigue.

### CT reporting

CT scans were initially reported in routine clinical practice by a mixed pool of radiologists, including specialist and non-specialist reporters. Original radiology reports were retrospectively categorised as indicating stable disease, disease progression or no opinion. All scans were independently reviewed by an ILD-MDT thoracic radiologist using the same categories; this expert assessment served as the reference standard.

### Quantitative assessment

Automated quantitative analysis was performed retrospectively using contextflow ADVANCE Chest CT (contextflow GmbH, Vienna, Austria).^8^ Both the original reporting radiologist and the ILD MDT radiologist were blinded to assessment. Following automated lung segmentation, a machine-learning algorithm quantified the relative volumes of multiple lung parenchymal textures, including honeycombing and reticular pattern. Absolute change in qCT features, expressed as a percentage of total lung volume, was calculated between the two CT scans. Absolute changes in qCT metrics were adjusted by time interval to give annualised change in qCT. Cases in which automated processing failed were excluded.

### Cohort stratification

After applying inclusion and exclusion criteria, the final cohort was split at the patient level into derivation (training) and internal validation cohorts using a stratified random approach, stratified by ILD-MDT thoracic radiologist outcome (stable vs progressive). A 70/30 split was used for derivation and validation cohorts, respectively.

### qCT threshold derivation

In the derivation cohort, the primary qCT metric was defined as the absolute change in combined honeycombing and reticular pattern between CT scans. Youden’s index was used to identify an optimal qCT threshold for identifying disease progression as defined by the ILD-MDT thoracic radiologist. The threshold chosen aimed to maximise sensitivity to reduce false negative cases.

### Internal validation

In the validation cohort, qCT was evaluated as a decision-support tool to assist non-specialist radiologists. The qCT output was permitted only to upgrade cases to progression where the original radiologist reported stable disease or expressed no opinion and was not permitted to downgrade original assessments. This approach was intended to maximise identification of disease progression while preventing potentially clinically inappropriate downgrading based solely on automated assessment.

Diagnostic correctness of unaided and AI-assisted interpretation was assessed against the ILD-MDT thoracic radiologist reference standard. Paired correctness was compared using a two-sided McNemar’s test.

### Study registration

This study was registered as a service evaluation with the Royal United Hospitals Bath NHS Foundation Trust and did not require external ethical approval. A STARD Checklist is available in online supplemental information 1.

### Statistics

Statistical analyses were performed using Python (version 3.13.5) within JupyterLab using pandas, NumPy and SciPy. Continuous variables are summarised as mean (SD) and categorical variables as counts (%). Paired differences in classification accuracy between unaided and AI-assisted interpretations were assessed using McNemar’s test for paired binary outcomes, with original reports categorised as ‘no comment’ considered incorrect. Group comparisons were undertaken using T-Test, Kruskal–Wallis and Mann–Whitney U tests.

## Results

### Cohort selection

502 patients were assessed in the ILD-MDT between 01/01/2023 and 31/12/2024. 127/502 (25.3%) of cases met inclusion and exclusion criteria (figure 1). 89 patients were in the derivation and 38 patients in the validation cohort. Patient demographics, diagnosis and pulmonary function testing of these patients are found in table 1. Table 1 demonstrates the proportions of patients meeting INBUILD criteria for PPF in the 24 months prior to ILD-MDT.

**Figure 1:**
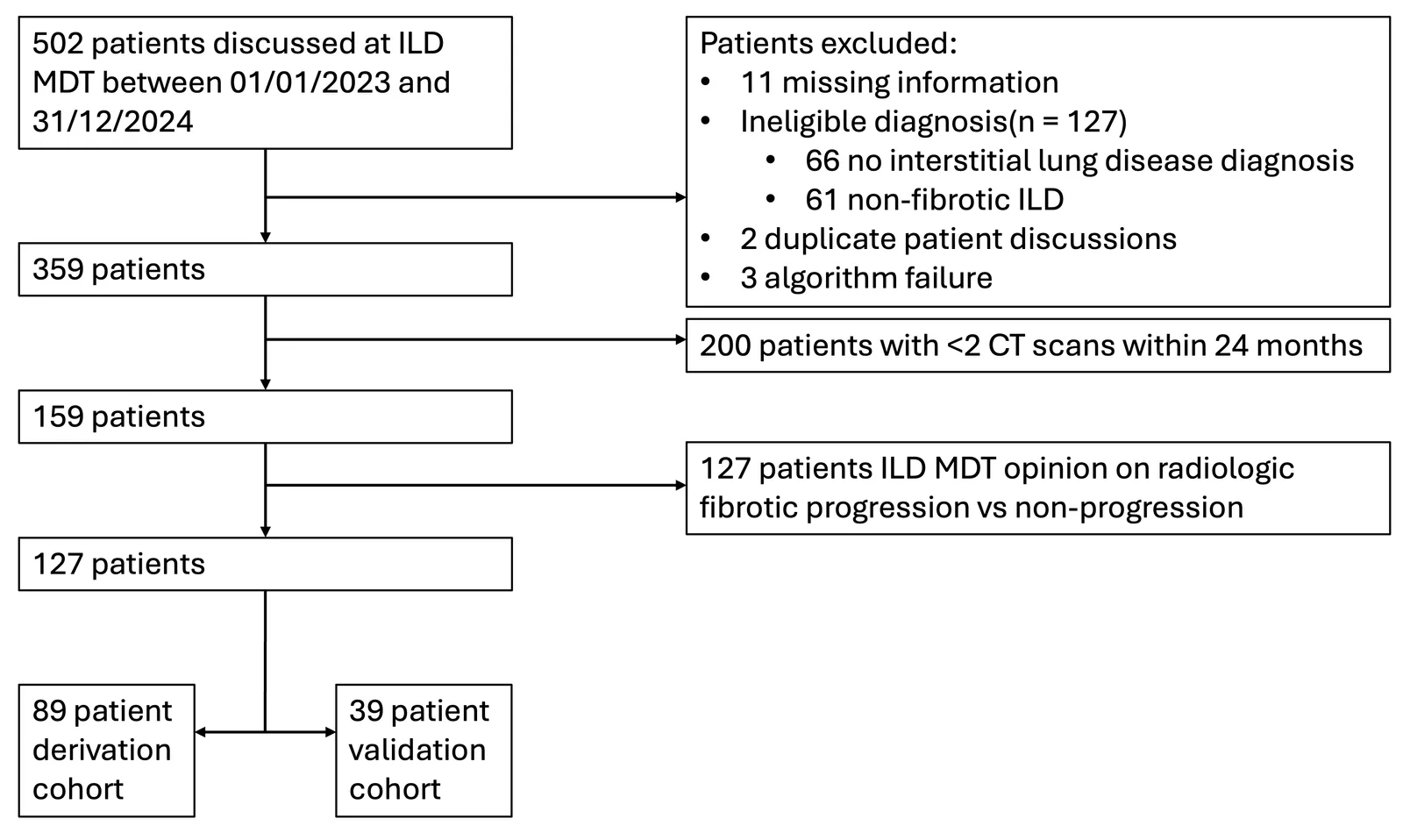
Consort diagram demonstrating cohort selection and stratification into derivation and validation cohorts. ILD, interstitial lung disease; MDT, multidisciplinary team.

**Table 1:** Baseline features of derivation and validation cohorts

Variable		Derivation	Validation	Comparison
N		89	38	n/a
Male sex n, (%)		54 (60.7%)	16 (42.1%)	p=0.08
Age at MDT (years), mean (SD)		71.9 (10.4)	72.0 (9.4)	p=0.94
Diagnosis				p=0.84
Unclassifiable ILD		38 (42.7%)	15 (39.5%)	
Idiopathic pulmonary fibrosis		16 (18.0%)	5 (13.2%)	
Connective tissue disease, ILD		14 (15.7%)	7 (18.4%)	
Idiopathic interstitial pneumonia		8 (9.0%)	6 (15.8%)	
Fibrotic hypersensitivity pneumonitis		4 (4.5%)	1 (2.6%)	
Interstitial lung abnormality		4 (4.5%)	1 (2.6%)	
Rheumatoid-arthritis ILD		3 (3.4%)	3 (7.9%)	
ANCA-associated vasculitis ILD		1 (1.1%)	0 (0.0%)	
Asbestosis		1 (1.1%)	0 (0.0%)	
ILD-MDT radiologist determined disease progression				
Stable		41 (46.1%)	17 (44.7%)	n/a
Progression		48 (53.9%)	21 (55.3%)	n/a
FVC % at time of MDT, mean (SD)		89.0 (43.4)	80.8 (21.2)	p=0.16
Missing FVC % data (n)		3	1	
TLCO % at time of MDT, mean (SD)		51.8 (15.2)	52.4 (21.1)	p=0.88
Missing TLCO data (n)		4	2	
Median (IQR) duration between CT scans (days)		352 (362)	319 (357)	n/a
Number of patients meeting INBUILD Criteria for PPF				
>10% Relative decline in FVC over 24 months	Progression	16 (18%)	5 (13.2%)	n/a
	No progression	36 (40.4%)	12 (31.6%)	n/a
	Insufficient data	37 (41.6%)	21 (55.3%)	n/a
5–10% Relative decline in FVC and progressive symptoms	Progression	5 (5.6%)	3 (7.9%)	n/a
	No progression	47 (52.8%)	14 (36.8%)	n/a
	Insufficient data	37 (41.6%)	21 (55.3%)	n/a
5%–10% Relative decline in FVC and progressive changes on CT	Progression	6 (6.7%)	1 (2.6%)	n/a
	No progression	46 (51.7%	16 (42.1%)	n/a
	Insufficient data	37 (41.6%)	21 (55.3%)	n/a
Progressive changes on CT and progressive symptoms	Progression	28 (31.5%)	11 (28.9%)	n/a
	No progression	50 (56.2%)	19 (50.0%)	n/a
	Insufficient data	11 (12.%)	8 (21.1%)	n/a

P values were calculated using Welch’s t-test for continuous variables and Pearson’s χ² test for categorical variables. Cohorts were stratified by progression status and therefore this variable was not compared between groups. ILD diagnosis was compared between cohorts using Pearson’s χ² test of independence across all diagnostic categories.

FVC, forced vital capacity; ILD, Interstitial lung disease,; MDT, multidisciplinary team; TLCO, transfer capacity for carbon monoxide.

### Original vs ILD-MDT radiologist diagnosis

In the derivation cohort cases were originally defined as progression (30/89, 33.7%), stable (44/89, 49.4%) or no comment (30/89, 33.7%). Of those described as stable or with no comment, 24/59 (40.7%) of cases were upgraded to disease progression by an expert thoracic radiologist at ILD MDT (figure 2A).

**Figure 2:**
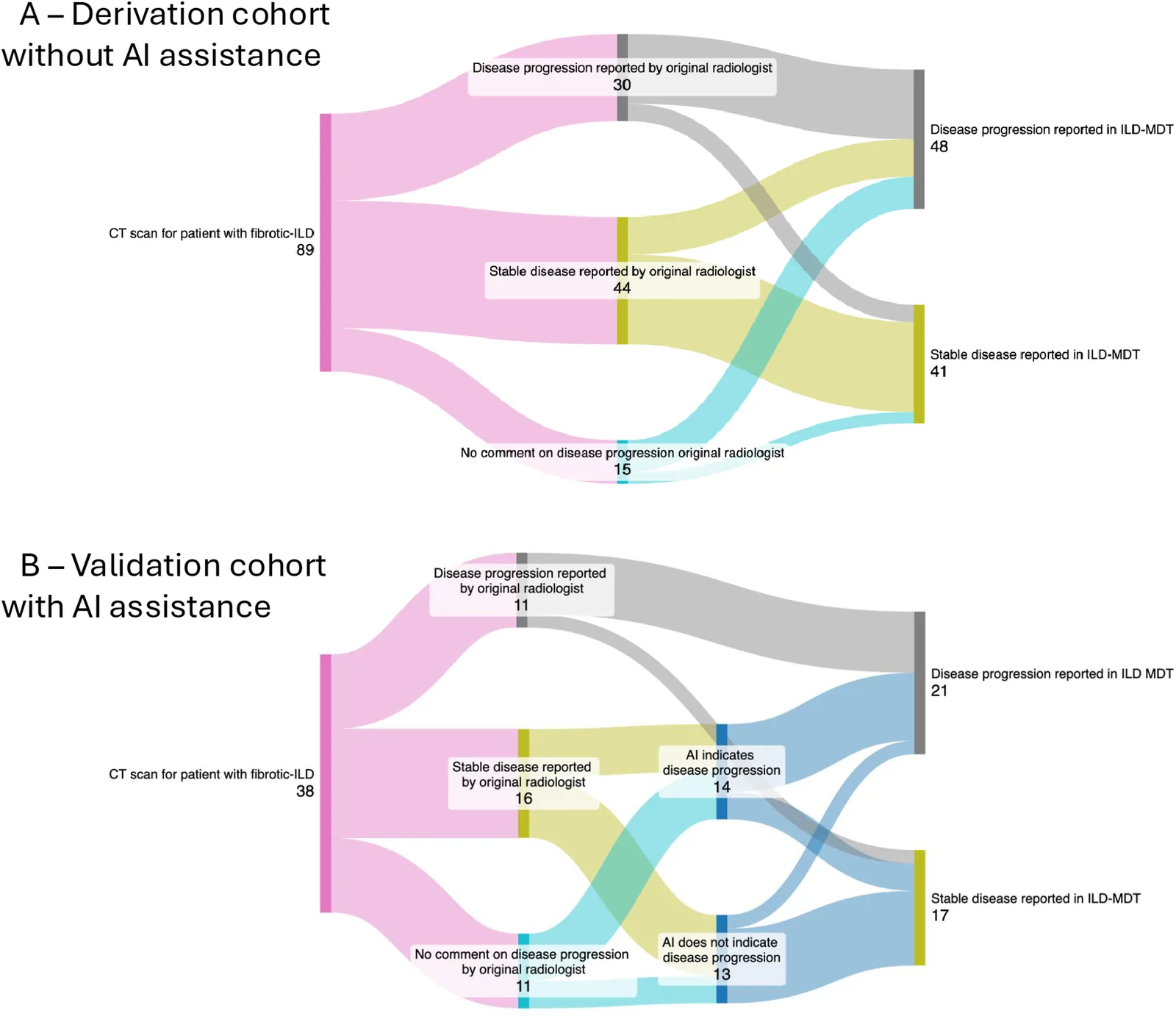
(A) Sankey diagram demonstrating discrepancy between original reporting radiologist and ILD-MDT expert radiologist defined fibrotic interstitial lung disease progression in derivation cohort. (B) Sankey diagram demonstrating the use of artificial intelligence (AI) to assist the identification of progressive pulmonary fibrosis for patients with fibrotic interstitial lung disease in validation cohort. ILD, interstitial lung disease; MDT, multidisciplinary team.

### qCT threshold derivation

The mean and SD of the Δ qCT features between ILD-MDT expert thoracic radiologist defined disease stability or progression are seen in table 2. There was no significant difference in mean Δ qCT features between progressive and stable groups. Of note, there was on average an increase in all parameters in both groups during the study.

**Table 2:** Mean and SD of absolute change in qCT feature between ILD-MDT expert thoracic radiologist defined disease stability versus progression

qCT feature	Absolute δ in qCT feature between CT scans (mean %, SD)		Two-sided t-test P value
	Progressive	Stable	
Total lung abnormality	+5.15 (17.1)	+3.47 (12.9)	0.60
Honeycombing	+1.26 (2.04)	+0.77 (3.03)	0.38
Reticulation	+0.65 (2.3)	+0.08 (2.64)	0.28
Honeycombing + reticulation	+1.91 (3.42)	+0.85 (4.83)	0.24

ILD-MDT, interstitial lung disease-multidisciplinary team.

Optimal thresholds for identifying ILD-MDT thoracic radiologist–defined disease progression were derived for each qCT feature using Youden’s index, with the corresponding area under the receiver operating characteristic curve reported (table 3).^5^

**Table 3:** Optimum threshold derived from Youden’s index for the identification of ILD-MDT expert thoracic radiologist defined disease progression for four different qCT features

qCT feature	Threshold (% change)	Sensitivity	Specificity	Area under the curve
Total lung abnormality	+7.37	0.44	0.80	0.564
Honeycombing	+0.33	0.54	0.80	0.654
Reticulation	+0.48	0.54	0.63	0.609
Honeycombing+reticulation	+0.39	0.69	0.54	0.636

ILD-MDT, interstitial lung disease-multidisciplinary team.

The combination of honeycombing and reticulation was selected as offering the highest sensitivity to enable the optimal identification of patients with PPF (cut-off value +0.39, sensitivity 0.69 and specificity 0.54).

### qCT-assisted pathway change

In the validation cohort cases were originally defined as progression (11/38, 28.9%), stable (16/38, 42.1%) or no comment (11/38, 28.9%). Of those described as stable or with no comment 12/27 (44.4%) of cases were upgraded to disease progression at ILD-MDT review. When the combined change in honeycombing and reticulation was applied to these cases, 10/12 (83.3%) cases were correctly identified as having disease progression by the qCT (figure 2B). These 10 cases with pathway changes included three unclassifiable ILD, three non-IPF IIP, two CTD-ILD and two patients with IPF. Furthermore, of these cases, five had originally been described as stable and five had no comment on disease progression.

For the McNemar analysis, original radiologist reports categorised as ‘no comment’ were considered incorrect. In the validation cohort, when the original radiologist assessment was correct (n=18), the AI-assisted assessment was also correct in 12 cases (66.7%). When the original radiologist assessment was incorrect (n=20), the AI-assisted assessment was correct in 15 cases (75.0%). McNemar’s test did not demonstrate a statistically significant difference in paired correctness between unaided and AI-assisted interpretation (χ² = 3.05, p=0.08).

### Annualised change in qCT

The median (IQR) number of days between CT scans was 352 (IQR 362 days) and 319 (IQR 357 days) for the derivation and validation cohorts. Given the wide range in CT scan interval, annualised change in qCT was assessed.

An annualised change in honeycombing + reticulation of +0.26 had a sensitivity and specificity of 0.71 and 0.51, respectively. In the 12 patients in the validation cohort incorrectly defined as stable or no comment, 10/12 (83.3%) were correctly identified as having disease progression. McNemar’s test did not meet statistical significance (p>0.05).

### PPF criteria and qCT change

Online supplemental tables 2 and 3 present the mean absolute and annualised changes in qCT features between the two analysed CT scans, stratified by INBUILD progression criteria assessed during the 2 years preceding ILD multidisciplinary team discussion. Across all analyses, there was little evidence of an association between qCT change and INBUILD-defined progression. The only statistically significant finding was a difference in absolute honeycombing change across groups defined by a 5%–10% relative decline in FVC with worsening respiratory symptoms (Kruskal–Wallis p=0.03). This finding was not replicated on pairwise comparison between progressors and non-progressors using the Mann–Whitney U test. No statistically significant associations were consistently observed across the derivation and validation cohorts in the annualised change analysis.

## Discussion

This study has demonstrated a proof-of-concept study for the use of artificial intelligence (AI) to assist the non-specialist radiologist to flag PPF for referral to the ILD-MDT. Radiological disease progression combined with either physiological or symptomatic disease progression is the the most common factor in the UK for the diagnosis of PPF leading to the initiation of nintedanib.^9^ As the use of CT becomes more common for the monitoring of fibrotic-ILD, there is an increased risk non-specialist radiologist availability might lead to inequity for the diagnosis of PPF and therefore access to appropriate treatment.^10^

The use of radiology AI tools to assist radiologists in complex disease interpretation is growing with demonstration of improved performance in mammography interpretation and chest radiograph reporting.^11 12^ The number of commercially available AI tools for the assessment of ILD is growing including Brainomix 360 e-Lung (www.brainomix.com/ild), Fibresolve (imvaria.com) and contextflow ADVANCE Chest CT (https://contextflow.com/). As these products are integrated into patient pathways there needs to be high-quality real-world validation studies to demonstrate patient and potentially health economic benefit. This study provides a proof of concept that could be developed prospectively to generate this evidence.

Studies have demonstrated that qCT can offer prognostic information of patients for fibrotic-ILD beyond what is offered by visual interpretation.^13^ However, this same study demonstrated that the R^2^ correlation between disease extent defined by visual radiology assessment vs qCT quantification was only 0.46. Varying degrees of concordance between visually- and qCT-defined PPF have previously been reported however these studies have not assessed the positioning of qCT into clinical pathways.^14 15^ This study demonstrates the potential placement of qCT in a real-world setting demonstrating feasibility for further prospective implementation studies.

This study supports the notion that visual interpretation is vital to identify non-classical features of disease progression. This is highlighted in the current study whereby an increase in all qCT features was noted in the group defined as stable by the specialist ILD MDT (table 2). This may reflect several factors. First, variability in serial qCT acquisition and analysis may introduce sufficient noise to generate detectable but clinically insignificant changes in qCT features. Second, visual assessment, even by expert thoracic radiologists, may lack the sensitivity to identify subtle but potentially clinically meaningful changes detected by quantitative CT analysis.

Importantly, the threshold selected to trigger specialist MDT review (+0.39 for combined honeycombing and reticulation) was lower than the mean increase observed among patients classified as radiologically stable by the ILD-MDT (+0.85). As the threshold was chosen to maximise sensitivity, some scans identified as potentially progressive by qCT were subsequently deemed stable on expert visual assessment. Longitudinal follow-up will be required to determine whether these individuals ultimately develop progressive disease, which would suggest that qCT detects early progression not yet apparent on visual interpretation.

A key potential advantage of qCT, as with home spirometry, is the earlier detection of PPF.^16^ However, direct evidence that earlier identification and treatment of PPF improves clinical outcomes is currently lacking, and prospective studies are needed to determine whether this translates into meaningful patient benefit.

In our study the proposed qCT feature of combined honeycombing and reticulation score will not identify other more subtle features of disease progression such as traction bronchial dilatation or loss of lobar volume.^17^ For this reason, the demonstrated approach of qCT+non-specialist radiologist to flag cases for specialist review, rather than using qCT to rule out a specialist review seems most valid.

It is noticeable that our cohorts had a significant proportion of unclassifiable-ILD. In the study design selected cases with <2 CT scans in 24 months were excluded. Patients with IPF typically have a limited number of CT scans as demonstration of progression is not required in the UK to initiate anti-fibrotic treatment. In the case of unclassifiable disease, the use of serial CT can not only help with diagnosis but can also detect progressive disease which may be eligible for anti-fibrotic treatment.

The role of qCT in identifying PPF should be considered alongside established progression criteria. Online supplemental tables 2 and 3 demonstrated no reproducible differences in qCT features between patients who did and did not meet INBUILD progression criteria, suggesting that qCT-assessed progression captures a distinct aspect of disease behaviour from visual radiological progression, physiological decline and worsening symptoms. However, this analysis was subject to significant missingness particularly in PFTs. Further work is needed to determine the clinical significance of qCT-assessed disease progression. The current study presents a theoretical pathway to highlight patients for review by expert thoracic radiologist in an ILD-MDT.

### Limitations

This study was limited by several factors; first, as a retrospective study, there was significant attrition from the original population to identify eligible patients. However, our intention had been to accurately identify a population undergoing serial CT scanning for the identification of PPF. An alternate study design in the future might prospectively test inter-observer agreement for disease progression between specialist and non-specialist radiologists and then incorporate AI assistance.^18 19^ Despite this, our study offers a real-world population as a proof of concept for development.

The study used both contrast and non-contrast scans to reflect real-world practice and sub-group analysis or stratification by contrast status was not possible due to study size. Future study should mandate protocolised non-contrast high resolution CT to reduce noise introduced by scan protocols.

The study only looked at specific outputs from the studied algorithm. There were other features not assessed in this study including ground glass opacification, consolidation, effusion, emphysema and pneumothorax. These were however analysed collectively in the total abnormalities analysis. Future study could investigate each qCT feature for their individual merits.

The study was unable to offer robust comparison with disease progression based on pulmonary function testing due to limited availability of adequate data. This is likely reflective of reduced PFT availability due to the lack of protocolised follow-up schedules for outpatients.

In our study the ILD-MDT radiologists were unblinded to the original reports. Despite this, there were significant proportion of cases who underwent pathway change following review and as such the influence of the original report is thought to be minimal. The original radiologist pool may have contained a small proportion of specialist thoracic radiologists. These data were unavailable at the time of analysis; however, the proportion of specialist reported scans is small at the studied institution.

The cohort selected in the study was of patients referred to ILD-MDT. There may have been cases not discussed in ILD-MDT where multiple CT scans were performed and not referred. These cases may be enriched for radiologist defined non-progressors.

The final validation cohort is likely limited by sample size leading to an insignificant McNemars analysis. However, the study still demonstrated the correction of 10/12 scans incorrectly labelled as stable disease or with no comment.

In addition, this was a single-centre study, and therefore both the derivation and validation cohorts reflect local imaging protocols, scanner characteristics, and reporting practices. While this design is appropriate for proof-of-concept development and internal validation, it may limit generalisability to other institutions. Multicentre prospective validation will be required to assess robustness across different scanners, acquisition parameters and patient populations. Furthermore, the use of data splitting to develop predictive models has limitations which will need to be addressed with future external validation.^20^

Finally, the threshold values used to define progression were derived and evaluated within the same institutional dataset. Although internal validation was performed using an independent cohort, the stability and transferability of these thresholds across centres remain unproven. Prospective multicentre evaluation will be necessary to confirm the clinical utility of this threshold and to determine whether site-specific calibration is required.

## Conclusion

This proof of concept study has shown that non-specialist radiologist assisted by AI can reduce the number of errors made in the identification of PPF. Prospective, multicentre validation is required to understand and maximise potential patient benefit.

## Supplementary material

10.1136/bmjresp-2026-004217 online supplemental file 1

10.1136/bmjresp-2026-004217 online supplemental file 2

## Data Availability

Data are available upon reasonable request.
