# Supplementary material for: Automated quantitative analysis can support the identification of progressive pulmonary fibrosis in fibrotic interstitial lung disease
Source: BMJ Open Respir Res. 2026 Oct 20;13(1):e004217. doi: 10.1136/bmjresp-2026-004217 (PMC13684211; doi:10.1136/bmjresp-2026-004217)
Supplement: Supplementary file 2 [file bmjresp-13-1-s002.docx]

| **Cohort** | **INBUILD Criteria** | **Criteria met** | **Mean (SD) change in Honeycombing + Reticulation** | **Mean (SD) change in Honeycombing** | **Mean (SD) change in Reticulation** | **Mean (SD) change in Total Lung Abnormality** |
| --- | --- | --- | --- | --- | --- | --- |
| Derivation | >10% relative decline in FVC | No progression | 2.20 (5.06) | 1.38 (3.28) | 0.82 (2.75) | 5.80 (16.11) |
| Derivation |  | Progression | 0.65 (3.64) | 1.24 (2.13) | -0.59 (2.71) | 6.85 (16.56) |
| Derivation |  | Insufficient data | 1.00 (3.22) | 0.60 (1.76) | 0.40 (1.95) | 1.91 (13.83) |
| Derivation | 5–10% decline in FVC + worsening respiratory symptoms | No progression | 1.77 (4.33) | 1.31 (2.85) | 0.46 (2.62) | 5.38 (15.91) |
| Derivation |  | Progression | 1.28 (8.02) | 1.60 (4.20) | -0.32 (4.45) | 13.13 (17.97) |
| Derivation |  | Insufficient data | 1.00 (3.22) | 0.60 (1.76) | 0.40 (1.95) | 1.91 (13.83) |
| Derivation | 5–10% decline in FVC + progressive changes on CT | No progression | 1.41 (4.61) | 1.23 (2.90) | 0.18 (2.85) | 4.96 (15.99) |
| Derivation |  | Progression | 4.15 (5.03) | 2.16 (3.56) | 1.99 (1.67) | 15.07 (15.18) |
| Derivation |  | Insufficient data | 1.00 (3.22) | 0.60 (1.76) | 0.40 (1.95) | 1.91 (13.83) |
| Derivation | Progressive changes on CT and worsening symptoms | No progression | 1.36 (4.65) | 0.87 (2.77) | 0.49 (2.82) | 1.34 (15.02) |
| Derivation |  | Progression | 1.99 (3.48) | 1.54 (2.43) | 0.45 (1.63) | 9.96 (16.03) |
| Derivation |  | Insufficient data | 0.26 (3.05) | 0.48 (1.41) | -0.22 (2.64) | 3.94 (10.71) |
| Validation | >10% relative decline in FVC | No progression | 2.82 (3.66) | 1.23 (1.79) | 1.59 (3.50) | -2.81 (10.28) |
| Validation |  | Progression | 1.30 (3.86) | 0.46 (1.09) | 0.83 (3.06) | 3.37 (4.92) |
| Validation |  | Insufficient data | 1.04 (3.08) | 0.54 (2.03) | 0.50 (1.86) | -1.39 (13.69) |
| Validation | 5–10% decline in FVC + worsening respiratory symptoms | No progression | 1.95 (3.63) | 0.48 (1.01) | 1.47 (2.89) | -1.24 (6.42) |
| Validation |  | Progression | 4.34 (3.84) | 3.48 (1.84) | 0.86 (5.68) | 0.16 (20.66) |
| Validation |  | Insufficient data | 1.04 (3.08) | 0.54 (2.03) | 0.50 (1.86) | -1.39 (13.69) |
| Validation | 5–10% decline in FVC + progressive changes on CT | No progression | 2.52 (3.74) | 0.72 (1.16) | 1.80 (2.86) | -1.94 (8.75) |
| Validation |  | Progression | 0.01 (NA) | 5.58 (NA) | -5.58 (NA) | 14.22 (NA) |
| Validation |  | Insufficient data | 1.04 (3.08) | 0.54 (2.03) | 0.50 (1.86) | -1.39 (13.69) |
| Validation | Progressive changes on CT and worsening symptoms | No progression | 0.99 (2.76) | 0.22 (1.21) | 0.76 (2.30) | -1.12 (14.86) |
| Validation |  | Progression | 2.89 (4.43) | 1.79 (2.79) | 1.10 (3.47) | -0.36 (8.50) |
| Validation |  | Insufficient data | 1.45 (3.00) | 0.56 (0.88) | 0.89 (2.20) | -2.62 (7.79) |

Supplementary Table 2. Absolute change in quantitative CT measures over 2 years according to INBUILD progression criteria in the derivation and validation cohorts.

| **Cohort** | **INBUILD Criteria** | **Criteria met** | **Mean (SD) annualised change in Honeycombing + Reticulation** | **Mean (SD) annualised change in Honeycombing** | **Mean (SD) annualised change in Reticulation** | **Mean (SD) annualised change in Total Lung Abnormality** |
| --- | --- | --- | --- | --- | --- | --- |
| Derivation | >10% relative decline in FVC | No progression | 2.42 (4.63) | 1.28 (2.78) | 1.14 (2.87) | 4.39 (16.49) |
| Derivation |  | Progression | -1.91 (10.50) | 1.14 (1.47) | -3.06 (10.76) | 5.39 (17.38) |
| Derivation |  | Insufficient data | 4.16 (13.11) | 2.18 (8.14) | 1.98 (6.22) | 7.64 (46.13) |
| Derivation | 5-10% decline in FVC + worsening respiratory symptoms | No progression | 1.11 (7.38) | 1.25 (2.43) | -0.14 (6.90) | 4.14 (17.04) |
| Derivation |  | Progression | 0.86 (5.20) | 1.10 (2.81) | -0.24 (2.73) | 9.96 (11.74) |
| Derivation |  | Insufficient data | 4.16 (13.11) | 2.18 (8.14) | 1.98 (6.22) | 7.64 (46.13) |
| Derivation | 5-10% decline in FVC + progressive changes on CT | No progression | 0.71 (7.42) | 1.14 (2.44) | -0.43 (6.94) | 3.40 (16.68) |
| Derivation |  | Progression | 4.01 (3.94) | 1.99 (2.55) | 2.02 (1.99) | 14.68 (13.16) |
| Derivation |  | Insufficient data | 4.16 (13.11) | 2.18 (8.14) | 1.98 (6.22) | 7.64 (46.13) |
| Derivation | Progressive changes on CT and worsening symptoms | No progression | 2.99 (12.86) | 1.84 (7.02) | 1.15 (8.32) | -0.93 (28.95) |
| Derivation |  | Progression | 2.22 (5.03) | 1.73 (3.13) | 0.48 (2.71) | 16.06 (36.02) |
| Derivation |  | Insufficient data | -0.12 (3.58) | 0.37 (1.14) | -0.49 (2.96) | 11.26 (30.33) |
| Validation | >10% relative decline in FVC | No progression | 1.18 (3.39) | 0.94 (2.08) | 0.24 (3.70) | -3.80 (13.38) |
| Validation |  | Progression | 0.05 (3.04) | 0.31 (0.81) | -0.26 (2.65) | 4.10 (6.18) |
| Validation |  | Insufficient data | 2.97 (14.77) | 2.31 (10.37) | 0.67 (6.50) | -1.77 (34.14) |
| Validation | 5-10% decline in FVC + worsening respiratory symptoms | No progression | 0.52 (3.41) | 0.22 (0.74) | 0.30 (3.01) | -2.52 (11.66) |
| Validation |  | Progression | 2.37 (2.11) | 3.24 (3.32) | -0.87 (5.41) | 3.40 (15.63) |
| Validation |  | Insufficient data | 2.97 (14.77) | 2.31 (10.37) | 0.67 (6.50) | -1.77 (34.14) |
| Validation | 5-10% decline in FVC + progressive changes on CT | No progression | 0.90 (3.34) | 0.36 (0.78) | 0.54 (2.89) | -2.69 (11.36) |
| Validation |  | Progression | 0.01 (NA) | 7.07 (NA) | -7.07 (NA) | 18.02 (NA) |
| Validation |  | Insufficient data | 2.97 (14.77) | 2.31 (10.37) | 0.67 (6.50) | -1.77 (34.14) |
| Validation | Progressive changes on CT and worsening symptoms | No progression | 0.20 (4.40) | 0.14 (1.86) | 0.05 (5.45) | -1.47 (32.08) |
| Validation |  | Progression | 5.95 (19.87) | 5.00 (14.07) | 0.96 (6.87) | 0.40 (24.41) |
| Validation |  | Insufficient data | 0.94 (2.20) | 0.44 (0.83) | 0.51 (1.47) | -4.82 (12.24) |

Supplementary Table 3. Annualised change in quantitative CT measures over 2 years according to INBUILD progression criteria in the derivation and validation cohorts.
